# Supplementary material for: Are carbon emissions trading and green financial instruments synergistic? -Comprehensive quantitative research based on content analysis
Source: PLoS One. 2024 Mar 7;19(3):e0298601. doi: 10.1371/journal.pone.0298601 (PMC10919601; doi:10.1371/journal.pone.0298601)
Supplement: S1 Data — (ZIP) [file pone.0298601.s001.zip › Data/地级市Z_F工作报告.zip!/╣π╬≈/╣≤╕█╩╨/└┤╫╘╣1⁄2╓┌║┼í╛┬φ┐╦╩2╛▌═°í┐_2019.docx]

各位代表：

现在，我代表市人民政府，向大会报告政府工作，请予审议，并请市政协各位委员和列席会议的同志提出意见。

一、2018年工作回顾

过去一年，在自治区党委、政府和市委的坚强领导下，在市人大及其常委会和市政协的监督支持下，在市中级人民法院和市人民检察院的司法保障下，我们坚持以习近平新时代中国特色社会主义思想为指导，深入贯彻党的十九大和十九届二中、三中全会精神，持续深入落实“三大定位”新使命和“五个扎实”新要求，认真贯彻中央、自治区和市委的决策部署，按照市委提出的“13446”工作思路，坚持“稳中求好、好中快进”发展主基调，牢固树立新发展理念，认真落实高质量发展要求，深化细化“六个年”活动，持续开展“大招商、招大商”活动，凝心聚力，开拓创新，真抓实干，经济社会全面发展、均衡发展，呈现出质量高、增速快、后劲足的良好态势，较好完成了市五届人大三次会议确定的目标任务。

（一）经济发展稳中向好、好中快进

地区生产总值1169.9亿元，增长10%，全区排名第二，连续三年实现逆势提速、高速增长；财政收入突破100亿元，达106.6亿元，增长18.4%，增速全区排名第一；固定资产投资增长19.8%，连续三年全区排名第一；居民人均可支配收入22175元，增长9%；主要经济指标增速均排名全区前列，全部超额完成年初确定的目标任务。

工业经济扩量提质。深化细化“工业提质增效年”活动，推动出台《广西战略性新兴产业城（贵港）三年行动计划（2019—2021年）》，制定《关于推动贵港工业高质量发展的决定》及支持企业做大做强相关奖励办法，推动工业高质量发展。全市规模以上工业总产值增长22.6%，规模以上工业增加值增长13.5%、全区排名第三。新兴产业加速集聚，产值增长37.2%，获自治区定位为广西战略性新兴产业城。工业投资增长55.7%，全区排名第一，获评为广西工业投资评价优秀单位；久久星专用车下线，众泰贵港基地君马乘用车即将下线，贵和科技、爱玛电动车等项目试产，荷美专用车获得整车生产资质，上马科技、柳钢大菱电动车获国家3C认证。传统产业提档升级，荣获“中国南方板材之都”称号，林板纸产业产值增长54%；纺织服装、建材等产业产值增速均超过20%。预计企业技改投资增长46.2%，全区排名第一；贵钢特种钢技改项目投产，华润（平南）绿色工厂入选工业和信息化部绿色制造名单。新入库规模以上工业企业127家，全区排名第一；贵钢集团入选中国制造业企业500强，5家企业入选“广西百强企业”。

农业现代化步伐加快。农林牧渔业总产值341.7亿元，增长5.5%。粮食总产量147.2万吨，桂平市、港南区、覃塘区获中央财政常规产粮大县奖励。现代特色农业示范区增点扩面提质升级，累计建成自治区级现代特色农业核心示范区13个。“中国硒港”影响力扩大，荣获“全国富硒农业示范基地”称号，新增自治区认证富硒农产品15个、广西名优富硒产品4个。成功创建桂平维军生态国家级稻渔综合种养示范区、平南石硖龙眼中国特色农产品优势区，港北农业科技园被认定为广西农业科技园区。新增市级以上农业产业化龙头企业22家、农民专业合作社698家、家庭农场130家。畜禽规模养殖场生态养殖占比75%，494家养殖场通过广西畜禽现代生态养殖认证。“双高”糖料蔗基地建设扎实推进。农作物耕种收综合机械化水平62.1%。

现代服务业优化升级。社会消费品零售总额551.2亿元，增长7.6%；万达广场和上亿国际汽车城开业，广西永泰跨境电商产业园开园，建成登龙桥农贸市场、蓝田村农贸市场，开工建设贵港易农国际农产品交易中心、贵港吾悦城市广场；成功举办第三届中国富硒农业发展大会、中国—东盟林木高峰论坛、新能源汽车展等活动。港口货物吞吐量突破7000万吨，增长10.8%；集装箱吞吐量30万标箱，增长35.1%；贵港进境粮食指定口岸通过国家验收，贵港口岸核心能力建设通过海关总署复核；建成国际贸易“单一窗口”，关检业务实现“一次申报”。实施“旅游强市”战略，全力打造特色旅游品牌，接待游客总人数增长28.1%，旅游总消费增长34.5%，荣获“全国森林旅游示范市”称号，桂平市被评为“广西特色旅游名县”；平南龚州生态园、雄森动物大世界、北帝山旅游区和荷美覃塘景区被评为4A级景区，新增四星级以上乡村旅游区（农家乐）6家、广西休闲农业与乡村旅游示范点5家，西山泉旅游度假区被评为自治区级旅游度假区；成功举办中国健身名山登山赛、中国旅游日（广西分会场）等旅游活动。金融服务实体经济能力进一步增强，金融机构贷款余额增长17.7%。餐饮、住宿、保险、快递、电信等服务业稳步发展。

项目建设成效明显。深化细化“项目建设效益年”活动，全力推动项目多开工、多竣工，早开工、早竣工，736个主要建设项目完成投资401亿元，其中新开工项目220个、竣工90个；中央预算内投资项目开工率全区排名第一，自治区层面统筹推进重大项目投资完成率全区排名第三。储备项目1788个，634个列入自治区重大项目三年滚动计划储备项目库，24个纳入国家发展和改革委PPP项目库，8个纳入财政部PPP项目库。项目建设用地保障得到加强，新增建设用地指标1.15万亩，盘活存量建设用地2.9万亩，完成土地征收5.3万亩。

县域经济整体跃升。实施乡村振兴三年行动计划，深化细化“县域经济发力年”活动，预计五个县市区地区生产总值增速均高于全区平均水平。桂平市被评为“广西科学发展先进县”，平南县、覃塘区、港南区被评为“广西科学发展进步县”。桂平市、平南县入选“西部百强县市”、被评为“广西健康产业发展示范县”。港北区主要经济指标增速在广西21个主城区中排名前列。

创新能力持续增强。新增国家授权专利232件，每万人口发明专利拥有量增长38.8%，增速全区排名第一。新增国家高新技术企业14家、国家级星创天地1家。贵港高新技术产业开发区获自治区政府批复设立，新增自治区级农业科技园区2家、创新小镇2家、院士工作站1家。完成科技成果转化项目25项，任务完成率连续两年全区排名第一。完成技术交易额13.5亿元，增长10倍，增速全区排名第一。实施“商标品牌”战略成效凸显，新增注册商标1840件，增长24%。“质量强市”战略深入推进，14个产品荣获“广西名牌”称号。广西新能源电动车质检中心投入使用。

（二）改革开放纵深推进

营商环境不断优化。荣获“2018中国营商环境质量十佳城市”和“2018年度中国十佳营商环境示范城市”称号，企业投资环境满意度连续两年全区排名第一。取消行政审批事项10项，调整行政许可事项41项。行政审批中介超市服务基本实现投资项目全覆盖，中介服务提速60%以上，服务收费降低20%以上。市本级政务办事1066项实现“最多跑一次”，79项实现“一次不用跑”。认真贯彻中央和自治区降低实体经济企业成本政策措施，减轻企业负担23亿元。深化细化“企业服务品牌年”活动，持续开展“市长服务企业接待日”“市政府服务接待日”活动，切实解决企业提出的问题。组建贵港市12345便民服务中心，对市民咨询投诉实行一个平台受理、多个渠道分办、统一口径反馈。

重点领域改革富有成效。改革组建工业投资集团、农业投资集团、文化旅游投资集团。推进11家经营类事业单位改革。河长制湖长制验收考评成绩全区排名第一。财政改革进一步深化，财政管理工作获自治区督查激励通报。推进西江农场土地管理综合改革，与广西农垦局签订了《贵港市规划利用广西农垦西江农场土地协议》。县级公立医院综合改革效果评价通过国家复核，国有林场改革通过自治区验收，农村土地承包经营权确权登记颁证工作经验在全区推广。机构改革稳步推进，安全生产、不动产登记、金融、供销等重点领域改革有序推进。

招商引资持续发力。新增签约项目278个，合同投资额887.8亿元，增长8.3%，其中亿元以上项目205个，合同投资额820.1亿元，平铝轻量化车厢、贵和科技、金奔腾汽车服务云、安铂瑞医疗、中化集团新型肥料等项目落户。产业链招商取得新进展，成功引进“新三篇”项目18个，合同投资额142.5亿元。新开工招商项目178个；实际利用内外资519.2亿元，增长14.9%，增速排全区前列。

对外交流合作进一步加强。外贸进出口总额26.7亿元，增长12%；加工贸易进出口总额9.7亿元，增长115%，增速全区排名第一；实际利用外资4002万美元，增长17%。加强与美国蒙特利公园市、墨西哥梅里达市、越南富寿省的交流交往；积极推动中华文化走出去，外派华文教师工作全区领先。

（三）城乡基础设施不断完善

城市功能品质得到提升。深化细化“城市发展跃升年”活动，全面启动城市总体规划修编，有序推进西江新城控制性详细规划和广西第二汽车生产基地规划编制工作。青云大桥全线贯通，高铁广场一期投入使用，贵港园博园开园，一级连线道路改造工程、西江大桥维修加固工程完工，郁江两岸综合治理工程、金港大道东段高架桥、北环西环路改造工程、城区河湖连通工程等项目扎实推进。完成中心城区6条支路“白改黑”。基本消除城区道路雨后重大内涝积水点。推进“公交都市”建设，提供多样化的公交服务。智慧城市建设走在全区前列，荣获2018中欧绿色和智慧城市“技术创新奖”，成功承办2018中国绿色智慧城市发展高峰会议；建成大数据平台、云计算中心和智慧城市管理中心，智慧党建、智慧市政、智慧消防、智慧教育、智慧交通、智慧政务、智慧环保上线试运行。深入开展文明城市、卫生城市、食品安全示范城市、森林城市、园林城市、生态文明建设示范城市“六城”创建工作，荣获“国家森林城市”称号，继续保持“广西卫生城市”称号；在自治区文明城市实地测评中，得分全区排名第一；食品安全质量水平持续提升，园林绿化美化景观提档升级，城市生态环境更加宜居，广大市民享受到更高品质的城市生活。成功举办第32届全国荷花展览、第十一届广西园林园艺博览会。城镇建设力度加大，4个特色小镇列入自治区特色小镇培育名单。常住人口城镇化率达49.5%。

交通网络建设加快。贵港至隆安高速公路、荔浦至玉林高速公路、覃塘至石卡产业园一级公路等项目顺利推进，西外环高速公路、“九路两桥”项目全面启动，江南工业园至桥圩、猫儿山至武乐、木格至寨圩等8条公路开工。苏湾作业区进港公路通车，武林港二期、苏湾作业区一期竣工，贵港航运枢纽二线船闸工程建设加快。苏湾作业区进港铁路支线和郁水作业区进港铁路支线纳入国家“十三五”港口集疏运系统建设方案。

农村基础设施明显改善。推进大藤峡水利枢纽、病险水库除险加固、农田水利工程和中小河流治理等水利项目建设，总投资达46.5亿元。新增恢复改善灌溉面积39.4万亩，新增高效节水灌溉面积5.1万亩。实施农村饮水安全巩固提升项目50个，受益人口15.7万人。实施水库移民新村项目45个，受益群众3万多人。“四好农村路”建设加快，新建、改扩建农村公路646公里，71.6%的乡镇通二级路，88.4%的行政村开通客运班线。投入3.6亿元改造农村电网，新建改造低电压台区2054个。

（四）生态环境得到改善

深入贯彻落实习近平生态文明思想，切实担负起生态环境保护的政治责任，出台全面加强生态环境保护“1+4”系列文件，制定工作考核办法，健全完善工作机制，层层压实责任，生态环境保护工作上新台阶。

污染防治成效初显。全力攻坚蓝天保卫战，开展大气污染防治“百日攻坚”行动，空气质量得到改善。积极开展清水行动，建成镇级污水处理厂10个，城西污水处理厂提标改造工程、城东污水处理厂项目、城区污水直排口截污工程加快推进；率先在全区完成市本级集中式饮用水水源地环境问题整改，主要江河水考核断面水质达标率100%，县级以上集中式饮用水水源地水质达到或优于Ⅲ类标准；城区黑臭水体治理和非法网箱养殖整治成效明显。扎实推进净土行动，没有突发土壤污染环境事故。认真整改中央环境保护督察组“回头看”反馈问题，及时解决了一批突出环境问题，清理整治“散乱污”企业808家，群众举报环境问题处理率100%。

节能减排取得新成绩。单位地区生产总值能耗下降3%，能耗总量控制在自治区下达目标任务内，规模以上万元工业增加值能耗下降10.3%。实行最严格水资源管理制度，落实水资源消耗总量和强度“双控”目标，全市用水总量、万元地区生产总值用水量、万元工业增加值用水量控制在自治区下达目标任务内。

“美丽贵港”乡村建设加快。实施“厕所革命”，新建改建旅游和市政厕所56座，改造农村厕所6万户。完成农村改厨6万户。实施农村环境综合整治项目40个，建成“清洁田园”生态田园示范片区143个。港北区荣获“自治区级生态县”称号，15个乡镇荣获“自治区级生态乡镇”称号，平南县镇隆镇富藏村荣获“全国生态文化村”称号。完成造林15.03万亩，森林覆盖率46.42%。

（五）民生福祉持续增进

加大民生投入，民生领域财政投入增长13.6%，支出占比达83.5%。以广西第一名的成绩入选中国地级市民生发展100强。

脱贫攻坚有新突破。深化细化“精准扶贫突破年”活动，预计111个贫困村、6.41万贫困人口脱贫摘帽，贫困发生率下降到2.8%。贫困人口建档立卡数据质量排名全区第一。在自治区扶贫开发成效考核中，桂平市获一等等次，平南县、港北区、港南区、覃塘区获二等等次。产业扶贫扩面增效，县级“5+2”特色产业覆盖率91%，建成105个就业扶贫车间，所有贫困村集体经济收入达3万元以上。易地扶贫搬迁综合进度全区排名第一，在全区率先完成“十三五”易地扶贫搬迁安置任务。建设村屯道路694.9公里，改造农村危房9686户。发放贫困户学生奖助学金1.1亿元、惠及贫困学生6.4万人次。农村最低生活保障标准提高12.5%。健康扶贫“198”工作落实到位，一站式保障系统全面建成；建档立卡贫困人口城乡居民基本医疗保险参保率100%，贫困人口住院费用报销比例90%以上。认真整改自治区巡视组扶贫领域机动式巡视反馈问题。

教育事业全面发展。建成中山小学、港南一小、桂平凤凰小学等6所小学和中山幼儿园、油麻中心幼儿园、国安瑶族乡甘雅幼儿园等6所幼儿园；开工建设荷城二中、港南三中、平南工业园初中、覃塘实验小学等27所学校；贵高新校区建设加快。学前三年毛入园率87%。五个县市区全部通过国家义务教育均衡发展督导评估。高中教学质量有新提升，高考每万人口本科上线率全区排名第一。高等教育迈出新步伐，广西工业学院项目开工，广西物流职业技术学院项目加快推进。职业教育、民办教育持续健康发展，其他各类教育取得新成绩。教育信息化工作再上新台阶，村完全小学以上学校校园网建成率94.7%，全区排名第一。

民生保障能力不断增强。城镇新增就业1.9万人，农村劳动力转移就业新增4.6万人，城镇登记失业率1.35%，比控制目标任务数低3.22个百分点；有效落实就业创业政策，获自治区督查激励通报。“五险”参保人数743万人次。企业、机关事业单位退休人员养老金月人均分别提高了4.1%和6%。建成市社会福利中心、康乐湾养老中心，创建示范性基层老年协会26家，港南区四季花田获“自治区养生养老小镇”称号。投入5亿多元，保障城乡低保对象、特困人员、残疾人、优抚对象、孤儿和老年人生活。防灾减灾工作得到加强，防汛抗旱工作扎实有效。保障性住房建设力度加大，新开工棚户区改造住房10874套。

文化事业更加繁荣。新建村级公共服务中心104个。“荷城”城市品牌、“和为贵”文化节影响力进一步扩大，大型历史题材话剧《压舱石》获第十届广西戏剧展演桂花铜奖，纪录片《大藤峡》在广西卫视和广东卫视播放，被列为自治区文艺精品。历史文化遗产得到保护传承，完成罗泊湾漪澜塔修缮，建成3个自治区级非物质文化遗产保护工作平台，港北壮族油纸伞制作技艺和桂平石龙壮族春牛舞入选自治区级非物质文化遗产代表性项目名录；5个村落入选中国传统村落名录；在第二届东盟地区论坛城市应急救援研讨班上，传承180多年的《平南大安消防节》得到了与会国家和国际组织的高度评价。在全区地级电视台率先启用高清播出系统。意识形态工作得到加强，社会主义核心价值观深入人心。黄大年纪念馆开馆，爱国主义教育广泛开展。

健康贵港建设加快。医疗卫生事业健康发展，实施卫生基础能力建设项目53个，市妇幼保健院新院投入使用。落实分级诊疗制度，医联体建设加快，基本实现大病不出县。基层卫生服务能力得到提升，17家乡镇卫生院荣获国家级“群众满意的乡镇卫生院”称号。全民健身活动广泛开展，成功举办国际马拉松赛、国际龙舟邀请赛、全国健美健身公开赛等重大赛事。

安全生产形势总体稳定。深入开展“落实企业安全生产主体责任”和“强监管、严执法”专项行动，深化安全隐患大排查大整治，没有发生重特大生产安全事故。在全区率先实行企业现场作业岗位安全生产信息公开告知制度。推行安全生产责任保险制度，企业安全生产责任险参保率全区排名第一。

庆祝自治区成立60周年活动圆满成功。出色完成广西壮族自治区成立60周年大庆各项任务。筹措87.7亿元，实施了社保惠民、健康惠民、教育惠民等33个大庆惠民实事项目。各项活动隆重热烈，俭朴务实，突出政治性，充分展示了我市民族团结、经济繁荣、社会进步、奋力跨越的新气象，得到中央代表团的充分肯定。

其他各项社会事业上新台阶。“平安贵港”建设深入推进，“维稳基石工程”深入实施，信访“三场硬仗、五大活动”取得较好成效；扫黑除恶专项斗争成效全区首次测评调查得分全区排名第一，社会治安形势明显好转，人民群众安全感满意度持续提升。普法依法治理工作深入开展，普法宣传走在全区前列，村居法律顾问实现全覆盖。宗教工作扎实开展。国防教育、国防动员、民兵调整改革、人民防空、军事设施保护和双拥优抚安置等工作迈出新步伐，创建自治区“双拥”模范城“十连冠”通过自治区考评验收。对台、侨务、统计、绩效、物价、编制、档案、地方志、应急管理、地震、公积金、粮食、机关事务管理、二轻联社等工作取得新成效，工会、共青团、妇联、科协、文联、残联、贸促会、社科联、工商联、红十字会和法学会等群团组织在促进经济社会发展中发挥积极作用，税务、海关、海事、农垦、邮政、水文、气象、烟草、盐业、石油化工、船舶检验、地质勘探、无线电管理等中区直各部门和驻贵部队、武警官兵、消防指战员为贵港经济社会发展做出新贡献。

（六）政府自身建设得到加强

深学真信笃用习近平新时代中国特色社会主义思想，推进“两学一做”学习教育常态化制度化，广大党员干部“四个意识”不断增强，“四个自信”更加坚定，始终做到坚决维护习近平总书记党中央的核心、全党的核心地位，维护党中央权威和集中统一领导。切实履行全面从严治党主体责任和党风廉政建设“一岗双责”，认真落实中央八项规定及其实施细则精神，持之以恒纠正“四风”，严格执行国务院“约法三章”。抓好自治区党委第七巡视组反馈意见整改。修订《贵港市人民政府工作规则》。坚持重大问题向市委请示报告，坚持重大事项向市人大常委会报告，向市政协通报，自觉接受监督，人大代表建议和政协提案办复率均为100%。加强法治政府建设，提请人大审议《贵港市烟花爆竹燃放管理条例（草案）》《贵港市城市道路管理条例（草案）》；全面推行政府法律顾问制度，强化行政执法监督工作，行政复议、行政应诉工作得到加强。完善公众参与、专家论证和政府决定相结合的决策机制。强化审计监督，狠抓督查落实，政府执行力和公信力不断提升。加强政务公开，主动公开各类政务信息。

各位代表！过去一年取得的成绩实属来之不易。这是习近平新时代中国特色社会主义思想科学指引的结果，是党中央、国务院和自治区党委、政府坚强领导和亲切关怀的结果，是市委统揽全局、科学决策的结果，是市人大及其常委会法律监督、工作监督和市政协民主监督的结果，是市中级人民法院和市人民检察院司法保障的结果，是全市各级各部门和全市各族人民团结奋斗的结果。在此，我代表市人民政府，向全市各族人民，向人大代表、政协委员，向人民法院和人民检察院，向各民主党派、工商联、无党派人士、各人民团体，向驻贵人民解放军、武警官兵、公安司法干警、消防指战员，向中区直驻贵各单位，向所有关心支持贵港发展的社会各界人士表示崇高的敬意和衷心的感谢！

我们也清醒地看到，我市经济社会发展中还存在不少困难和问题：高质量发展的基础还不够牢，新旧动能转换步伐还不够快；城市功能品质有待优化，城市综合管理水平有待提高；生态环境保护、民生领域还有不少短板，基本公共服务保障能力还不强，影响社会和谐稳定的风险点还有不少。一些政府工作人员的服务能力、担当意识还不够强，“四风”问题尤其是形式主义、官僚主义仍一定程度存在。我们一定要直面问题，敢于担当，锐意进取，尽心竭力做好工作，不断开创政府工作新局面。

二、2019年工作安排

2019年是新中国成立70周年，是全面建成小康社会关键之年，也是全面贯彻落实习近平总书记重要题词精神的第一年。做好今年工作意义重大，影响深远。我们要进一步坚定发展信心、保持战略定力，乘势而上、主动作为，奋力谱写新时代贵港发展新篇章。

今年政府工作总体要求是：坚持以习近平新时代中国特色社会主义思想为指导，深入贯彻党的十九大和十九届二中、三中全会精神以及中央庆祝改革开放40周年大会、中央经济工作会议、自治区党委十一届五次全会、全区经济工作会议精神，按照“建设壮美广西、共圆复兴梦想”的总目标总要求，持续深入落实“三大定位”新使命和“五个扎实”新要求，统筹推进“五位一体”总体布局，协调推进“四个全面”战略布局，围绕建设六“壮”六“美”新广西，按照市委提出的“13446”工作思路，坚持新发展理念和“稳中求好、好中快进”发展主基调，深入实施“五提升一决胜”六个年活动，统筹推进稳增长、促改革、调结构、惠民生、防风险工作，保持经济持续健康发展和社会大局稳定，为全面建成小康社会收官打下决定性基础，迈出打造新时代实力活力魅力给力新贵港，加快建成西江流域核心港口和新兴工业城市的坚实步伐，开启建设壮美广西、共圆复兴梦想新征程，以优异成绩庆祝中华人民共和国成立70周年。

根据总体要求，今年经济和社会发展的主要预期目标是：地区生产总值增长9%，财政收入增长10%，固定资产投资增长15%，规模以上工业增加值增长13%，社会消费品零售总额增长10%，外贸进出口总值增长10.5%，万元地区生产总值能耗下降2%，主要污染物排放总量控制在自治区下达目标内，居民人均可支配收入增长9%，居民消费价格指数涨幅控制在3%左右，城镇登记失业率控制在4.3%以内，城镇新增就业1.7万人，常住人口城镇化率49.9%，农村贫困人口减少7.46万人。

为实现上述目标，我们将重点做好以下几方面工作：

（一）构建现代产业体系，推动经济高质量发展

推动新旧动能转换和融合发展，调整优化产业结构，提高经济质量效益。

发展壮大工业经济。深入开展“工业发展提升年”活动，年内规模以上工业总产值增长23%以上。围绕建成战略性新兴产业城目标，大力发展新能源汽车、新一代信息技术、生物医药、智能装备制造、精细化工、新材料等产业，加快推进华奥二期、平铝轻量化车厢、石药泰诺、振华通讯等重大工业项目，推动腾骏、赛尔康、嘉龙海杰、爱玛、达庆生物等企业发展壮大。优化升级传统产业，抓好桥圩·中国羽绒谷、金源生物扩建、平南科技生态纺织园、华润装配式建筑等重点项目，推动建材、木材加工、冶金、羽绒、服装等优势产业“二次创业”。完善园区基础设施和配套设施，规划医药产业园，提升园区承载力，年内园区规模以上工业总产值占全市规模以上工业总产值的70%以上。

提升改造现代服务业。提升改造贵港储运贸易物流园，加快推进苏湾（西江）现代物流园、美家居国际博览城二期等项目，新建或改造城区农贸市场3个以上。举办富硒农产品、新能源汽车等各种展会和促销活动，提高贵港产品品牌知名度和影响力。加快发展港口运输业，年内港口货物吞吐量增长5%以上。大力实施“旅游强市”战略，年内旅游接待总人数增长28%以上、旅游总消费增长35%以上。积极创建全域旅游示范区。整合全市旅游资源，引进大型文旅公司重点打造一批特色旅游景点景区，构建旅游联盟衔接机制。加快状元山、汉古伞庄等旅游项目建设，力争贵港园博园、平天山国家森林公园、九凌湖景区成功创建4A级景区，推进桂平西山5A级景区创建工作。加强汽车营地、旅游度假区、生态旅游示范区等旅游新业态品牌创建工作。

增强科技创新能力。实施高新技术企业倍增计划，高新技术企业保有量50家以上。完善科技孵化体系，新增科技创新平台30家以上；完成科技成果转化项目10个以上。激发各类主体创新活力，每万人口发明专利拥有量增长15%以上。深化市校合作，强化人才支撑，大力引进高端人才、紧缺人才和创新团队。实施“商标品牌”战略，争取有一批地方特色产品获准地理标志证明商标注册。实施“质量强市”战略，全面提升产品、工程、服务、产业和人居质量。

（二）实施乡村振兴战略，推动农业农村现代化

把乡村振兴战略作为新时代“三农”工作总抓手，巩固发展“三农”持续向好形势。

推动县域经济发展新跨越。深入开展“县域经济提升年”活动，大力实施县域经济发展三年行动计划，壮大县域经济综合实力，确保各县市区GDP增速均高于全区平均水平，力争2个县市区获评广西科学发展“先进县”或“进步县”。落实促进县域经济发展各项措施，支持各县市区抓好优势产业发展、产业集群培育、园区建设等工作。加强县域基础设施和公共服务设施建设，布局和建设一批强县富民的大项目、好项目。推进新型城镇化，加快覃塘荷美新区建设，推动桂平市、平南县县城扩容提质，打造一批各具特色的产业小镇、历史文化小镇、民俗风情小镇。

坚决打赢打好脱贫攻坚战。深入开展“精准扶贫决胜年”活动，紧扣“两不愁、三保障”，注重脱贫质量，年内实现67个贫困村、7.46万贫困人口脱贫摘帽。大力发展特色产业，实现每个贫困村都有现代特色农业示范点。壮大贫困村村级集体经济，确保全部贫困村村级集体经济收入达4万元以上。提升贫困地区基础设施水平，新建村屯道路300公里以上，实施农村饮水安全巩固提升项目20个以上，完成农村电网改造项目30个以上。完善教育医疗住房“三保障”体系，实现贫困村贫困户义务教育巩固率100%；落实健康扶贫各项政策，贫困人口住院费用实际报销比例90%以上，门诊慢性病报销比例80%以上，实现“一站式”信息交换和即时结算服务；完成贫困家庭危房改造7849户。强化智慧农业产业扶贫精准技术服务。统筹抓好就业扶贫、生态扶贫、旅游扶贫等精准扶贫工程。全力支持桂平市做好脱贫攻坚，确保桂平市在2019年底达到贫困县脱贫摘帽各项指标要求，实现稳定脱贫摘帽。

发展现代特色农业。紧抓粮食生产不放松，确保粮食总产量135万吨以上。抓好果蔬、茶叶、中药材、肉蛋鱼禽等特色优势种养，打造珠三角和粤港澳绿色农产品生产基地和配送中心；成立百香果育苗中心、研究院和协会，打造广西乃至全国百香果发展最优平台。实施现代特色农业示范区建设增点扩面提质行动，新建自治区级现代特色农业核心示范区2个以上，实现每个县市区都有示范区、每个乡镇都有示范园、每个乡镇60%以上的村都有示范点。实施富硒品牌提升行动，建成富硒优质稻产业基地6万亩以上，新增认证富硒农产品5个以上；举办和参加各种富硒展览交易会，打响贵港“中国硒港”品牌。依照市场化和农民自愿的原则，加快推进农村土地流转，发展多种形式适度规模经营，培育新型农业经营主体，新增农民专业合作社200家以上、家庭农场100家以上。创建一批粮食生产功能区、重要农产品生产保护区、特色农产品优势区和现代农业产业园、科技园、创业园。全面完成8万亩“双高”糖料蔗基地建设任务。发展生态养殖，畜禽规模养殖场生态养殖占比80%以上，抓好水库洁水养鱼、微循环流水养鱼、稻渔综合种养示范点建设。强化农机全程社会化服务，农作物耕种收综合机械化水平64%以上。

提升农产品深加工水平。做强制糖、林产品、粮食、羽绒、畜禽水产等加工业，壮大果蔬、中药材、茶叶、桑蚕等特色农产品加工业。发展富硒农产品加工业，规划建设富硒农产品深加工园区。培育农产品加工特色小镇和田园综合体。积极引进和培育农产品加工龙头企业，力争新增自治区级或国家级农业产业化龙头企业4家以上，加快形成县县有规模以上加工业、优势特色产业，有加工龙头企业带动的发展格局。鼓励企业打造全产业链，支持农民专业合作社发展农产品加工流通业。

完善农业农村基础设施。实施乡村风貌提升三年行动，开展实用性村庄规划，分类推进村庄整治建设，建成基本整治型村庄1990个、设施完善型村庄260个、精品示范型村庄28个。建设“四好农村路”，新建、改建农村公路500公里以上，90%以上的行政村开通客运班线；建成根竹至平天山、石牛水库至平天山、金田起义进入景区道路等公路，开工建设桥圩至东津、动界至寺面、桂平新垌至玉林北市等通乡二级公路，年内75%以上乡镇通二级路。抓好农田水利基础设施建设，新增恢复改善灌溉面积5万亩以上，完成6座小型病险水库除险加固，继续加大大藤峡水利枢纽工程建设力度。加快移民新村建设，实施水库移民基础设施项目100个以上。新建、改造配电台区1900个。升级改造供销社服务网络，构建上下贯通、安全高效的农村物流体系。

创新农村社会治理。推行“农事农办”“农事网办”，加快村级综合服务中心规范化、标准化建设。建设平安乡村，推进县乡村三级综治中心和农村“雪亮工程”建设，下沉社会治安防控力量，实施网格化服务管理，促进农村矛盾纠纷精准排查、精细化解。实施公民道德建设工程，开展移风易俗行动，引导农民向上向善、孝老爱亲、重义守信、勤俭持家，提高乡村社会文明程度。

（三）扩大改革开放，增强发展动力活力

深入学习贯彻习近平总书记在庆祝改革开放40周年大会上的重要讲话精神，落实供给侧结构性改革“巩固、增强、提升、畅通”八字方针，坚定不移深化改革，扩大开放。

优化营商环境。深入开展“营商环境提升年”活动，强力推进“354560”“减证便民”等措施落到实处，深化“一事通办”改革，全面建成政务服务“一张网”，实现90%以上的市县政务服务事项网上办理、90%以上的依申请政务服务事项“最多跑一次”。实施“证照分离”改革扩容提速，深化“多证合一、一照一码”改革，深入推进企业登记全程电子化。强化项目联合预审，剔除“一票否决”因素，大幅压缩工程建设项目审批时间。建立完善政企沟通、优化营商环境投诉举报和招商引资项目代办服务“三大平台”，打造外来投资服务“金字招牌”。持续开展“市长服务企业接待日”“市政府服务接待日”活动，切实解决企业、市民和基层一线反映的困难问题。

推进重点领域改革。深化国企国资改革，建立健全出资人监管权力和责任清单，推动国资监管从管资产向管资本转变；抓好混合所有制改革，增强国有经济活力。深化基本公共服务领域市以下财政事权和支出责任划分改革，全面实施预算绩效管理改革。稳步推进政府机构改革，优化政府机构设置和职能配置。探索建立林长制。统筹推进农村集体产权、水利、教育、司法、事业单位、社会保障、城市管理、农村投融资、供销社等领域改革。

扩大招商引资。围绕强龙头、补链条、聚集群，深入开展“招商引资提升年”活动，坚持招商引资“主官工程”，继续强化全民招商、重力招商，年内新增签约亿元项目150个以上，新开工招商项目160个以上，到位资金增长12%以上。坚持产业链招商，围绕现有产业，培育壮大产业集群，着力引进一批汽车、电子、现代农业、高端木业等项目。提高招商引资针对性，聚焦大健康、大数据、大物流、新制造、新材料、新能源等重点产业，着力引进一批有实力、有经验、有市场、有眼光的企业。以项目早开工、早竣工、早投产为首要目标，完善合同条款，加强项目服务，全面提高合同履约率、资金到位率和项目开工率。

做大做强民营经济。启动实施强优民营企业培育计划，创建民营经济示范市、县、乡（镇）和示范园区。落实国家和自治区减轻企业税费负担政策，细化配套惠企措施，切实降低企业要素成本。创造充足市场空间，扩大民营经济市场准入。拓宽融资渠道，解决民营企业融资难融资贵融资慢问题。建立完善行政执法监督制约机制，维护民营企业合法权益。构建“亲”“清”新型政商关系，加强民营企业家队伍建设，在全社会营造尊重企业家、关心企业家、支持企业家干事创业的浓厚氛围。

深化对外交流合作。做好贵钢集团、赛尔康电子、保利高塑胶、益嘉发服饰、金源化工等重点外贸企业服务工作，鼓励企业扩大外贸进出口。促进外商投资稳定增长，年内实际利用外资增长8%以上。深化与国际友城交流，扩大国际友城“朋友圈”，继续做好华文教育工作。积极参与珠江—西江经济带建设，加快建设粤桂黔高铁经济带合作试验区广西园贵港分园，主动融入“一带一路”、国际陆海贸易新通道、粤港澳大湾区、北部湾经济区，着力构建全方位开放新格局。

（四）加强城市建设管理，提升城市功能品质

坚持人民城市为人民，统筹规划、建设和管理，深入开展“城市品位提升年”活动，进一步提高城市发展持续性、宜居性。

规划建设西江新城。以抓好城市总体规划修编为龙头，高标准科学规划西江新城和广西第二汽车生产基地。围绕把新城建设成新时代生态文明典范城区的目标，统筹推进港口、产业园区和城市生活区三大建设，促进港产城互动融合发展。加快建设广西第二汽车生产基地、中国—东盟新能源电动车生产基地、西江保税物流中心（B型）；高标准建设贵港高新技术产业开发区，启动国家高新技术产业开发区创建工作。大力推进西江教育园区基础设施和广西工业学院、广西物流职业技术学院、贵高新校区等学校项目建设。

完善城市基础设施。开工建设同济大道、城市规划馆、南山公园改造工程、市民中心和郁江城区防洪项目，加快推进金港大道东段高架桥、西环北环路综合改造、江南大桥、城东大桥、高铁广场二期、郁江两岸综合治理工程、南湖整治工程、城区第二水源等项目。抓好道路设施、园林绿化等改造提升，基本消除道路坑洼、人行道路破损、绿化黄土裸露等现象。着力打通城区“断头路”。改造城区供水管网，提升城区供水能力。启动泸湾江取水点搬迁工作。推进海绵城市建设。加快同济大道、西南大道城市风貌和小街小巷改造。

加强城市精细化管理。深入开展创城工作，巩固提升国家森林城市、广西卫生城市，力争获得全国卫生城市提名和广西食品安全示范城市，全国园林城市、文明城市、生态文明建设示范城市创建工作取得新进展。强化城市网格化管理，实现城市管理在“网”上提速，公共服务在“格”中提效。规范城市道路开挖和地下管线建设行为，杜绝“拉链马路”现象。深入开展市容市貌专项整治行动，突出解决交通秩序混乱、乱摆乱卖、侵占城市道路人行道、黑车非法营运、广告乱贴乱挂、农贸市场脏乱差等群众反映强烈的问题。推进“公交都市”建设，增加公交线路和专线公交，逐步开行城乡公交。严厉打击“双违”行为，坚决遏制增量，逐步消化存量。

构建大交通格局。年内贵港至隆安高速公路建成通车，开工建设西外环高速、高铁站交通枢纽工程。继续推进“九路两桥”和泗灵至高峰、社步至麻垌、覃塘至石卡产业园等一批路网项目。完善港口基础设施，开工建设贵港至梧州3000吨级航道，加快推进贵港航运枢纽二线船闸工程，抓好苏湾作业区进港铁路支线和郁水作业区进港铁路支线前期工作。启动贵港蒙圩机场和贵港苏湾机场前期工作。

建设数字贵港。加快基础设施智慧化、特色智慧应用、数据交换共享及运用建设，推进县市区智慧城市建设。启动实施数字政府建设三年行动计划，完善智慧党建、智慧政务、智慧市政、智慧环保、智慧公安、智慧消防等平台。发展“互联网+医疗健康”“互联网+教育”“互联网+养老服务”，提升公共服务均等化、普惠化、便捷化水平。推动物联网、云计算、大数据等新一代信息技术创新应用，实现与城市经济社会发展深度融合。

（五）着力改善生态环境，努力建设美丽贵港

全面贯彻落实习近平生态文明思想，践行“绿水青山就是金山银山”理念，坚决打赢污染防治攻坚战，大力推进生态文明建设。

打好蓝天保卫战。深入开展大气污染防治三年攻坚作战行动。强化工地扬尘管控，实行建筑工地现场全部封闭围挡、全部湿法作业、场区道路全部硬化、渣土物料全部覆盖、在建楼体全部封闭、出入车辆全部清洗。强化散货运输管控，推行城区特定范围密闭化公司化散货运输。强化秸秆焚烧管控，推动秸秆资源化利用。强化城市餐饮业油烟排放和烟花爆竹限放管控，全面取缔城区露天夜宵烧烤。推进园区集中供热，整治“散乱污”企业，全面开展水泥、板材、砖瓦等行业综合整治，确保工业企业稳定达标排放。启动城区重污染企业搬迁或升级改造。

打好碧水保卫战。深入开展水污染防治三年攻坚作战行动，推动河长制湖长制见行动见实效，巩固提升主要江河考核断面水质达标率、饮用水水源地达标率。加快建设城东污水处理厂、江南城区污水主干管，改造提升江南污水处理厂。完善污水管网，实施清污分流，提升城区污水收集处理率。推进城区污水直排口截污工程建设，完成5条河道截污与生态治理，基本消除城区黑臭水体。完善农村污水处理设施，建成18座镇级污水处理厂。建设病死畜禽无害化处理中心，全面清理禁养区畜禽规模养殖场、专业户。强化畜禽养殖粪污资源化利用。实行最严格水资源管理制度，持续推进江河湖库“清四乱”。

打好净土保卫战。深入开展土壤污染防治三年攻坚作战行动，确保不发生土壤环境污染事故。开工建设餐厨垃圾、固体废物处置中心和桂平生活垃圾终端分类处理项目，建成桂平市、平南县医疗废物收集中转站。提高工业固体废弃物综合利用率。加强重点行业企业用地土壤环境调查，强化重点区域、重点企业地块土壤监测、隐患排查和整治，不断提升土壤质量。

推动绿色低碳发展。深入实施生态经济重点工程，加快生态产业、生态基础设施、生态环境治理、生态城乡建设等领域重点项目建设。大力发展新能源、节能环保等产业，培育一批绿色工厂和绿色制造示范企业。扩大充电桩建设覆盖范围，推广新能源在公交、物流等车辆中的应用。提高环境准入门槛，严格控制新上高耗能、高排放项目。优化工业布局，推进园区循环化改造，出台相关优惠政策，鼓励、引导污染企业主动搬迁入园。开展全民绿色行动，倡导简约适度、绿色低碳的生活方式。

加强生态环境监管。落实领导干部生态文明建设责任制，严格执行“党政同责、一岗双责”。全面完成中央环境保护督察“回头看”反馈问题整改。严格执行考核评价和责任追究制度，认真落实环保信用评价、信息强制性披露、严惩重罚等制度。健全网格化环境监管体系，加强区域联防联治，严厉打击环境违法违规行为。

（六）保障和改善民生，着力增进群众获得感

坚持以人民为中心，把增进人民福祉、促进人的全面发展作为一切工作的出发点和落脚点，持续发展民生事业。

办好人民满意教育。建成东湖小学等6所小学，改扩建江南中学、达开高中、民族中学等学校，增加学位1万个以上；新开工建设荷城三中等3所中学、5所小学和3所特殊教育学校。继续实施学前教育行动计划，规范小区配套幼儿园建设使用，新增自治区认定多元普惠性幼儿园80所，学前三年毛入园率88%以上。抓好义务教育工作，义务教育巩固率95%以上。扩大高中教育规模，高中阶段毛入学率89%以上。抓好广西工业学院、广西物流职业技术学院办学申报工作。办好职业教育，鼓励社会力量依法兴办教育，重视发展民族教育、特殊教育、继续教育等各类教育。加强师德师风建设，培养高素质教师队伍。弘扬尊师重教的社会风尚，提高教师政治地位、社会地位、职业地位。

提高社会保障能力。实施就业优先政策，开展职业技能培训，加大创业扶持和就业援助，解决好高校毕业生、农民工、退役军人等群体就业。全面实施全民参保计划，深化社保“一门式”经办服务改革。强化对困难群体的生活兜底保障，城乡低保标准提高10%以上。做好被征地农民社会保障工作。发展居家、社区和互助式养老，推进医养结合。加强社会救助保障，健全社会救助体系，支持公益慈善事业发展。加强残疾康复服务。营造尊重妇女、关爱儿童、尊重老人，爱护残疾人的良好风尚。继续实施三年棚改攻坚计划，年内开工4300套以上。

建设健康贵港。完成贵港中心血站搬迁，推进贵港市东晖医院建设，启动贵港市第四人民医院、市疾病控制预防中心新大楼和覃塘区第二人民医院、民族医院、妇幼保健院项目。实施基层医疗卫生机构能力建设行动计划，年内所有乡镇卫生院基础设施达到国家标准。加快县域医共体建设，实现县域医共体乡镇卫生院全覆盖。加强医疗卫生人才队伍建设，75%的乡镇卫生院有2名以上全科医生，70%以上的乡村医生具有中职以上学历。传承发展中医药、壮瑶医药事业，支持社会办医。落实全面两孩政策配套措施。实施体育惠民工程，多渠道增加全民健身场所和设施，创建“健康贵港·休闲运动示范点”30个，积极申办国际国内大型体育赛事，加快发展体育产业。

发展文化事业。培育和弘扬社会主义核心价值观，提高公民素质和社会文明程度。加强文化遗产保护利用，规划建设贵港自然博物馆，加快推进南江古码头二期维修工程和大南门城楼修缮保护工程。抓好文化惠民工程，新建村级公共服务中心105个。广泛开展群众喜闻乐见的文化活动，提升“和为贵”文化节、荷花展、荷城大舞台“周周演”等文化品牌。坚持以人民为中心的创作导向，推出更多反映贵港新时代发展的文化艺术精品。加快广播电视村村通向户户通升级。推进全民阅读，共建书香贵港。

统筹发展其他各项社会事业。深入开展民族团结进步活动，支持民族地区发展。积极探索符合贵港实际的军民融合深度发展路径与模式，深化全民国防教育，做好新时代国防动员、后备力量、人民防空、双拥共建等工作，加强退役军人服务管理，维护军人军属合法权益，巩固军政军民团结，确保实现全国“双拥模范城”九连冠。加强社会信用体系建设。抓好档案、统计、公积金、粮食、物价、地方志、地震、绩效、二轻联社、机关事务管理、对台和侨务等工作，充分发挥工会、共青团、妇联、科协、文联、残联、贸促会、社科联、工商联、红十字会和法学会等群团组织作用，支持海关、海事、税务、农垦、邮政、通信、水文、气象、石油化工、烟草、电力、金融、盐业、船舶检验、地质勘探、无线电管理等中区直部门工作。

（七）打好防范化解重大风险攻坚战，确保社会和谐稳定

聚焦重点领域，防范和化解经济社会发展中的风险挑战。

防范金融债务风险。完善市县两级金融监管体系，打好防范化解金融风险攻坚战，严厉打击非法集资等违法违规金融活动，守住不发生系统性金融风险的底线。防范化解债务风险，依法合规举债。积极培植财源，增强可偿债综合财力。完善重大项目决策机制，加强财政承受能力评估论证，用好用足地方政府专项债券资金。清理财政借垫款，盘活沉淀资金，降低财政支付风险。

防范公共安全风险。深化“平安贵港”建设，加强社会治安综合治理，创新推进“维稳基石工程”，加强政法基础设施建设，加快立体化社会治安防控体系建设，持续开展扫黑除恶专项斗争，强化反恐维稳工作，严厉打击电信网络新型违法犯罪和黄赌毒、盗抢骗等人民群众反映强烈的各类违法犯罪行为。完善社会矛盾纠纷多元化解机制，创新信访工作方式，依法及时解决群众合理诉求。

防范安全生产风险。严格落实安全生产责任制，加大交通运输、建筑施工、消防火灾、非煤矿山、危险化学品、烟花爆竹、粉尘涉爆等重点行业领域风险防控和隐患排查治理，强化安全监管执法，加大监督检查和失责追责力度，坚决遏制重特大生产安全事故；完善突发事件应急体系，加强应急管理和救援能力建设，推动应急指挥系统标准化，科学妥善处置各类突发事件。

三、加强政府自身建设

各位代表！新时代对政府工作提出了新要求，我们必须按照习近平总书记提出的信念过硬、政治过硬、责任过硬、能力过硬、作风过硬要求，坚持担当为要、实干为本、发展为重、奋斗为荣，全力打造人民满意的服务型政府、法治政府和廉洁政府。

旗帜鲜明讲政治。坚持以习近平新时代中国特色社会主义思想武装头脑、指导实践、推动工作，精心组织开展“不忘初心、牢记使命”主题教育，提升政府系统广大党员干部的政治觉悟和政治能力，牢固树立“四个意识”，切实增强“四个自信”，坚决做到“两个维护”，确保中央、自治区和市委的各项决策部署不折不扣地贯彻落实。严格执行新形势下党内政治生活若干准则，进一步严肃政治纪律和政治规矩。持续推进自治区党委第七巡视组反馈意见整改落实。

坚持依法行政。推进法治建设，严格遵守宪法和法律，依照法定权限和法定程序行使权力、履行职责，把政府工作全面纳入法治轨道。坚持重大问题向市委请示报告，重大事项向市人大常委会报告，向市政协通报。坚决执行市人大及其常委会各项决议决定，自觉接受人大法律监督、工作监督，积极支持政协履行政治协商、民主监督、参政议政职能，认真办理人大代表建议和政协提案，主动接受社会监督。落实重大行政决策程序、“三重一大”事项决策机制。加强政府信息公开，主动回应社会关切。

强化实干担当。认真贯彻落实进一步激励广大干部新时代新担当新作为有关要求，大力弘扬新时代敢干、实干、苦干、善干“四干”新作风，坚持实字当头、干字为先，直面问题、说到做到，按照目标明确、措施明确、阶段性目标明确、责任人明确的要求，以“钉钉子”精神确保各项工作高标准推进、高质量完成。坚持问题导向，解难题、破瓶颈，做到及时主动发现问题、及时主动解决问题、及时主动协调问题、及时主动报告问题。强化督促检查，抓好责任落实。认真贯彻容错纠错机制，旗帜鲜明为敢于担当、踏实做事、不谋私利的干部撑腰鼓劲；坚决整治工作落实不力、效能低下、敷衍塞责等“慵懒散”行为。

建设廉洁政府。坚持以全面从严治党统领政府各项工作，扎实做好政府系统党风廉政建设和反腐败工作。坚决贯彻落实中央八项规定及其实施细则精神，驰而不息反“四风”，坚决破除形式主义、官僚主义。加强公共资金、国有资产、国有资源和领导干部经济责任审计监督，加大对权力运行的规范和制约。健全工程建设、土地出让、政府采购、产权交易等重点领域腐败风险防控机制，深化扶贫领域腐败和作风问题专项治理，坚决查处损害群众利益的问题。

各位代表！风劲帆满图新志，砥砺奋进正当时。让我们更加紧密地团结在以习近平同志为核心的党中央周围，坚持以习近平新时代中国特色社会主义思想为指引，在自治区党委、政府和市委的坚强领导下，开拓创新，真抓实干，一张蓝图绘到底，咬定青山不放松，不达目的不罢休，全力打造新时代实力活力魅力给力新贵港，加快建成西江流域核心港口和新兴工业城市，为建设壮美广西、共圆复兴梦想作出新的更大贡献，以优异成绩庆祝中华人民共和国成立70周年！
